# Treatment by Electricity

**Published:** 1894-09

**Authors:** 


TREATMENT BY ELECTRICITY; or, Electro-therapeutics, being a new System of Treatment introduced into India by Nondo Lal Ghose, L. M. S., Late Teacher of Medicine, Midwifery, Diseases of Women and Children, Patna, Dacca, and Campbell Medical Schools. Published by L. V. Mitter & Co., 27 Upper Circular Road, Calcutta, India.

This is a little pamphlet of sixty-four pages, setting forth the advantages of electricity in various diseases. Unfortunately it contains no details of procedures, being little more than a catalogue of the doctor's successes. Hence, it is not so interesting, nor is it so instructive as he might easily have made it.

